# Inspecting and unbending surgical needle holders

**Published:** 2023-12-01

**Authors:** Ismael Cordero

**Affiliations:** 1 Clinical Engineer, Philadelphia, USA.

*First published in Issue 81, 2013*

A needle holder, also called a needle driver (Figure 1), is made from stainless steel and is used to hold a suturing needle during surgical procedures.

**Figure 1:**
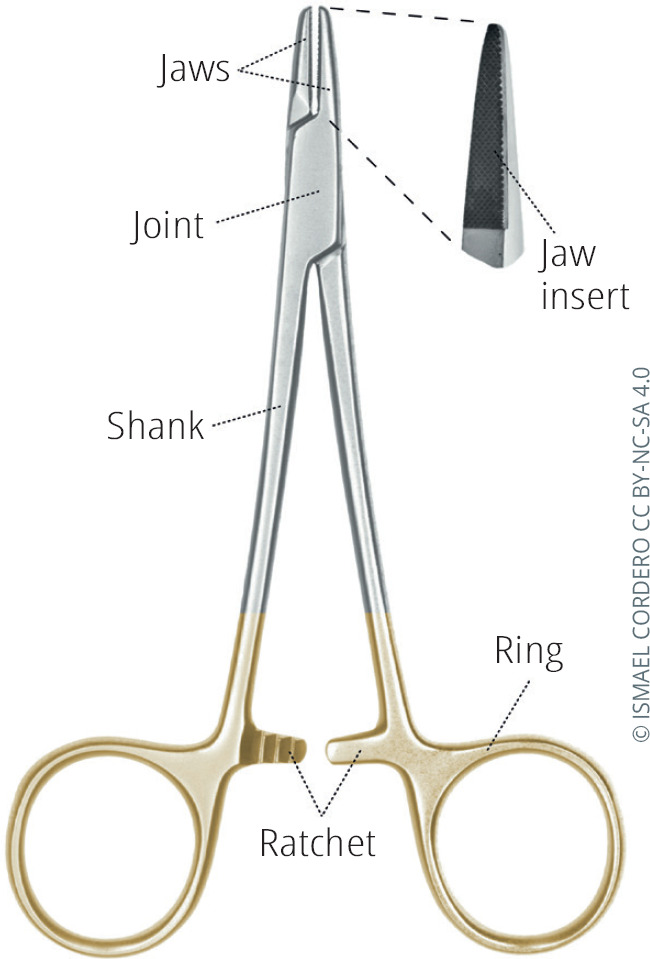
The main parts of a needle holder. The handles consist of a shank, a ring, and a ratchet mechanism that locks the needle in place.

To maintain a firm grip on the needle, the jaws have textured patterns either etched directly on the stainless steel or on a replaceable tungsten carbide insert, which grips the suture needle more precisely and wears out much slower than stainless steel. Needle holders with tungsten carbide inserts normally have gold-plated rings.

A needle holder must be matched to the needle size for which it is intended.

## Postoperative care

Open the needle holder by separating the ratchet. Prevent blood from drying onto the instrument by soaking it in an enzymatic solution. Alternatively, place a moist towel, saturated with water, over it within 20 minutes of use.

## Inspection and testing

A needle holder should be able to hold a hair on the back of your hand. If not, it is not functioning properly. With use, the jaw surfaces will wear out and stop making full contact, which affects their grip. Bends and cracks can also develop on the jaws and other parts of the needle holder.

It is important to inspect needle holders after each procedure and before sterilising them. Use a bright lamp and a magnifying glass or microscope to check for any of the following flaws.

- **Bent or worn jaws.** When the needle holder is held up to a bright light in the closed position, no light should shine through between the jaw surfaces. If the light only shines through a small portion of the jaws, either the jaw or the jaw insert is worn out. A worn jaw insert must be replaced by the manufacturer or a qualified vendor. If the jaw surface is worn (Figure 2), the entire needle holder must be replaced. If the light shines through a significant portion of the surface (Figure 3), one of the jaws is probably bent. Follow the procedure described later in this article to correct it.
- **Cracks in the jaws or joint.** Even small cracks compromise the integrity of the instrument. This means it should be sent to the vendor or manufacturer for repair.
- **Cracks in the jaw inserts.** The majority of insert damage occurs at the tips. The insert must be replaced if cracks are seen. If the tip of an insert looks and feels significantly less coarse than the rest of the insert, it should be replaced.
- **Rust and stains.** In order to determine whether a brown or orange discoloration is rust or a stain, rub a pencil eraser aggressively over part of the discoloration. If the discoloration cannot be removed and if there are pit marks, then it is rust and requires soaking in a rust removal solution and/or brushing carefully with a brass brush. If it can be removed and the metal underneath is smooth, then it is a stain and it can be removed by soaking in a stain removal solution.
- **Loose joint.**Open the instrument, grab one ring handle in each hand and gently push one handle up and down. There should be some give-and-take in the instrument, but if it feels too loose it should be repaired.
- **Poor ratchet fit.** Check that the jaw tips close in the first ratchet position and that the entire jaw closes in the third ratchet position. If a needle held in the jaws of a needle holder can be easily turned by hand with the instrument locked in the second ratchet position, repair is needed.

**Figure:**
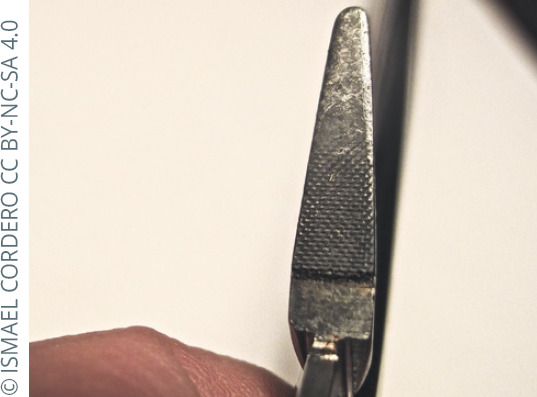
Figure 2

**Figure:**
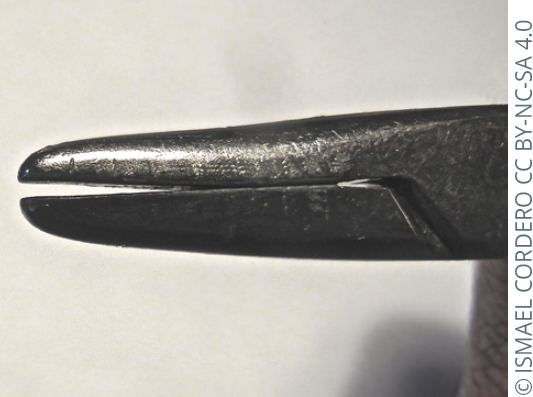
Figure 3

## Preparing for sterilisation

If any dried blood or discoloration is discovered on the needle holder, the instrument must be cleaned before being sterilised. Needle holders should always be sterilised with the ratchets disengaged.

## Correcting bent instruments

Bent needle holders can sometimes be corrected using a pair of flat-tipped pliers using the steps below. (**Note:** these procedures should not be used for needle holders with tungsten carbide inserts since they are brittle and can fracture easily.)

1. Close the needle holder, and look at it from the side. If you notice that the jaw tips are not aligned (Figure 4), then at least one tip is bent and you can try to straighten it with flat-tipped pliers (Figure 5). If it is not obvious which tip is bent, you can take turns bending both tips so that they align. **Note:** do not use too much force; bend the tips little by little.
2. Close the needle holders completely and hold them against a light. If light shines through the jaw surfaces (Figure 3) then you will need to bend one or both of the jaws towards each other.
3. If the ratchets do not hold anymore, bend the handles towards each other.
4. After unbending, test the needle holder by grabbing a hair on the back of your hand – the hair should not slip out.

**Figure:**
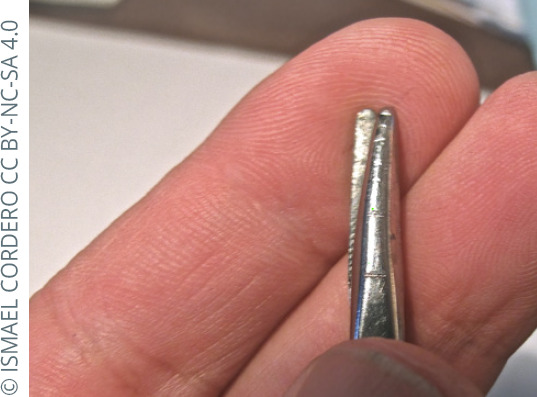
Figure 4

**Figure:**
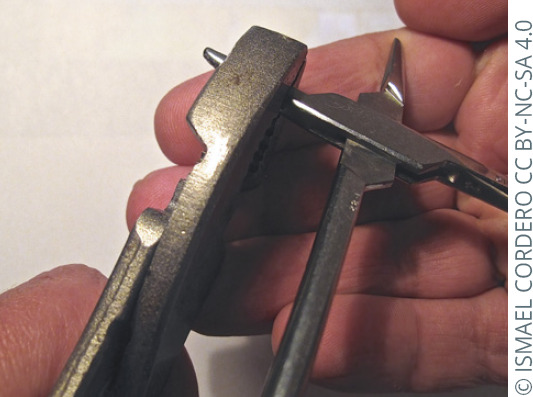
Figure 5
